# Value-based evaluation of dialysis versus conservative care in older patients with advanced chronic kidney disease: a cohort study

**DOI:** 10.1186/s12882-018-1004-4

**Published:** 2018-08-16

**Authors:** Wouter R. Verberne, Janneke Dijkers, Johannes C. Kelder, Anthonius B. M. Geers, Wilbert T. Jellema, Hieronymus H. Vincent, Johannes J. M. van Delden, Willem Jan W. Bos

**Affiliations:** 1 Department of Internal Medicine, St Antonius Hospital, Koekoekslaan 1, 3435 CM, Utrecht Nieuwegein, The Netherlands; 2 Department of Clinical Epidemiology and Medical Statistics, St Antonius Hospital, Nieuwegein, Utrecht The Netherlands; 3 University Medical Centre Utrecht, Julius Centre for Health Sciences and Primary Care, Utrecht, The Netherlands; 4 Department of Internal Medicine, Leiden University Medical Center, Leiden, Zuid-Holland The Netherlands

**Keywords:** Aged, Chronic kidney failure, End-stage renal disease (ESRD), Renal dialysis, Conservative treatment

## Abstract

**Background:**

Conservative care is argued to be a reasonable treatment alternative for dialysis in older patients with advanced chronic kidney disease (CKD). However, comparisons are scarce and generally focus on survival only. Comparative data on more patient-relevant outcomes are needed to truly foster shared decision-making on an individual level, and cost comparison is needed to assess value of care.

**Methods:**

We conducted a retrospective observational single-center cohort study in 366 patients aged ≥70 years with advanced CKD, who chose dialysis (*n* = 240) or conservative care (*n* = 126) after careful counselling by a multidisciplinary team in a non-academic teaching hospital in The Netherlands. Using a value-based health care approach (value = outcomes/cost): survival, health-related quality of life—cross-sectionally assessed with the Kidney Disease Quality of Life Short Form™—treatment burden, and treatment costs were evaluated.

**Results:**

The overall survival benefit of patients on a dialysis pathway compared with patients on conservative care diminished or lost significance in patients aged ≥80 years or with severe comorbidity. There were no differences between patients managed conservatively and dialysis patients on physical and mental health summary scores (all *P* > 0.1). Patients on conservative care had 352.7 hospital free days per year versus 282.7 in patients on a dialysis pathway, calculated from treatment decision (adjusted incidence rate ratio: 1.15, 95% confidence interval: 1.09 to 1.21, *P* <  0.001). Annual treatment costs were lower in patients on conservative care (adjusted cost ratio: 0.43, 95% confidence interval: 0.28 to 0.67, *P* <  0.001).

**Conclusions:**

In this study, conservative care is shown to be a viable treatment option in older patients with advanced CKD, particularly in the oldest old and those with severe comorbidity. By achieving similar outcomes at lower treatment burden and treatment costs, value was generated for older patients choosing conservative care and society.

**Electronic supplementary material:**

The online version of this article (10.1186/s12882-018-1004-4) contains supplementary material, which is available to authorized users.

## Background

In recent years, the number of older patients with advanced chronic kidney disease (CKD) has increased [1, 2]. As age is no longer seen as contraindication, dialysis treatment in older patients has become an established practice. The majority of dialysis patients is older than 65 years in countries like the United States, the United Kingdom, and The Netherlands nowadays [2–4]. Many older patients with advanced CKD have multiple comorbidities, are frail, and have increased dependency [5]. Dialysis has not always shown to benefit these patients in terms of survival, although evidence is still limited [6–9]. This has raised concerns about the suitability of dialysis—an intensive and expensive treatment—in this setting.

Conservative care (CC) is argued to be a reasonable alternative. In general, CC entails ongoing multidisciplinary care including all types of interventions as needed, though without dialysis [10]. Main goal is preservation of quality of life with adequate symptom control, instead of life prolongation per se. Estimates indicate that up to 15% of CKD patients choose to forego dialysis and prefer to be managed conservatively [11–13].

Shared decision-making has been recommended to align treatment plan with the patient’s values and preferences [14]. However, current decision-making is hampered by limited data on outcomes [10, 15, 16]. Most previous studies focused on survival only. To truly foster decision-making, data on more patient-relevant outcomes are needed [10, 17–19]. We evaluated survival, health-related quality of life (HRQOL), and treatment burden in older patients choosing dialysis or CC. This study is an extension of a previous survival analysis [9]. Treatment costs were assessed to evaluate value of care. This evaluation is based on the concept of value-based health care, in which value of delivered care is defined as the benefits on health outcomes achieved per monetary spent (value = outcomes/cost) [20, 21]. Using this value-based perspective, and by involving patients, our aim was to determine whether CC is a reasonable treatment option compared to dialysis for older kidney patients and society.

## Methods

### Study population

We identified a retrospective cohort of older patients with stage 4/5 CKD who received nephrology care in our non-academic teaching hospital between October 31, 2004 to May 1, 2016. Patients were included if they had chosen to be treated with dialysis or CC, and if aged ≥70 years at treatment decision. Patients needing immediate start of dialysis at presentation were excluded. As part of standard care, a shared decision-making process on treatment plan was initiated when renal function—determined as estimated glomerular filtration rate (eGFR)—dropped < 20 mL/min/1.73m^2^. A multidisciplinary team consisting of nephrologists, renal nurses, social workers and dieticians carefully counselled patients about possible treatment pathways including CC. In patients choosing haemodialysis (HD) or peritoneal dialysis (PD), dialysis treatment was prepared and initiated once needed. The dialysis group was defined as all who chose dialysis. Hence, the “dialysis group” also includes patients who chose dialysis but have not yet started dialysis at the end of study, and patients who died before initiation. In patients choosing CC, medical treatment and multidisciplinary care were continued. The study was approved by the local research ethics committee.

### Demographic and clinical data

Baseline data collected from electronic medical records included date of birth, sex, primary renal diagnosis according to the European Renal Association–Dialysis and Transplantation Association’s codes, comorbidities, height and weight to calculate body mass index, serum albumin level, and C-reactive protein level. Comorbidities were scored according to the Davies comorbidity score, based on the presence of seven comorbid conditions producing three risk groups (see Additional file 1) [22]. Renal function—measured as eGFR with the four-point Modification of Diet in Renal Disease formula [23]—was collected at treatment decision, and when eGFR consistently dropped < 20, < 15, and < 10 mL/min/1.73m^2^. Date of death was recorded after verification in the population register.

### HRQOL assessment

All included patients alive in 2015 and 2016 were asked to participate in a cross-sectional assessment of HRQOL. Exclusion criteria were mental incapacitation or language problems of such severity that the informed consent procedure and/or questionnaire could not be completed. The validated Dutch version of the Kidney Disease Quality of Life Short Form (KDQOL-SF™) was used [24, 25]. KDQOL-SF™ captures both generic and kidney disease-specific domains (see Additional file 1). Questionnaires were self-completed or interviewer-administered. Physical Component Summary (PCS) and Mental Component Summary (MCS) scores were calculated.

### Treatment burden

The outcomes on treatment burden included number of outpatient visits, admissions, in-hospital days, in-center haemodialysis days, and hospital free days as summary measure, comprising all medical specialties in our hospital. Data were collected from electronic medical records. Outcomes were assessed from treatment decision or start of dialysis, until death or end of study. Patients who were lost to follow-up were excluded. Outcomes were converted into annual incidence rates to adjust for differences in follow-up length, using total number of events as numerator and total follow-up time in years as denominator [26]. The annual number of hospital free days was estimated using the formula: 365.25 – (annual number of outpatient visits + in-hospital days + in-center haemodialysis days). In-center haemodialysis days were not counted on in-hospital days to prevent duplications. Further details are available in Additional file 1.

### Treatment costs

Treatment costs were defined as costs incurred through outpatient and inpatient care delivered by all medical specialties. Data were derived from the hospital’s financial administration. Due to changes in their system, cost data were reliably available from January 1, 2008. Treatment costs were assessed from treatment decision or start of dialysis, until death or end of study. Cost rates were annualised to adjust for follow-up length.

### Statistical analysis

Analyses were performed according to original treatment choice and dialysis modality. Patient characteristics were compared using descriptive statistics.

Survival was calculated using the Kaplan-Meier method, with assessment of differences using the log-rank test. Four starting points were used: treatment decision, eGFR < 20, < 15, and < 10 mL/min/1.73m^2^. Adjusted multivariable Cox regression analysis was performed to determine independent predictors of survival, considering: age, sex, primary renal diagnosis, Davies comorbidity score, eGFR at treatment decision, and treatment pathway. The statistically significant and near-significant variables in univariable analysis were used to construct Cox multiple regression models. Backward elimination was used to include only significant predictors of survival in the final model; hence, primary renal diagnosis and eGFR at treatment decision were excluded. Residuals and influential points were checked.

We compared HRQOL between CC patients, patients not yet started on dialysis, and patients started on dialysis. To test differences, students t-tests were used in PCS and MCS, and Mann-Whitney U tests in domain scores. Adjusted multiple linear regression analyses were performed on PCS and MCS to evaluate the association with treatment pathway, adjusting for: age, sex, Davies comorbidity score, and way of administration (self or by interviewer).

Incidence rate ratios were estimated to test differences in treatment burden outcomes, using generalised linear regression models with negative binomial distribution—as data were not normally distributed and overdispersed. Incidence rate ratios are interpreted similarly as odds ratios [26]. Adjustment variables were age, sex, Davies comorbidity score, and eGFR.

We report the mean annual treatment costs—recommended as most informative measure for cost data [27]—although cost data were not normally distributed. Negative binomial regression was used to assess differences by estimating the cost ratio. Adjustment variables were age, sex, Davies comorbidity score, and eGFR. Sensitivity analysis was performed—as recommended [28]—to test best model fit using generalised linear regression models with log-gamma and Poisson distribution.

A *P* value < 0.05 was considered statistically significant. Statistical analyses were performed using IBM SPSS 24.0.

### Patient involvement

In collaboration with the Dutch Kidney Patients Association, two patient representatives and a policy adviser of the Association were involved in designing the study. A systematic evaluation of our research protocol was performed using a guideline developed by the Association. Together with the Association, we also organized a group discussion with six patient representatives to interpret the results from patients’ perspectives.

## Results

**Fig. 1:**
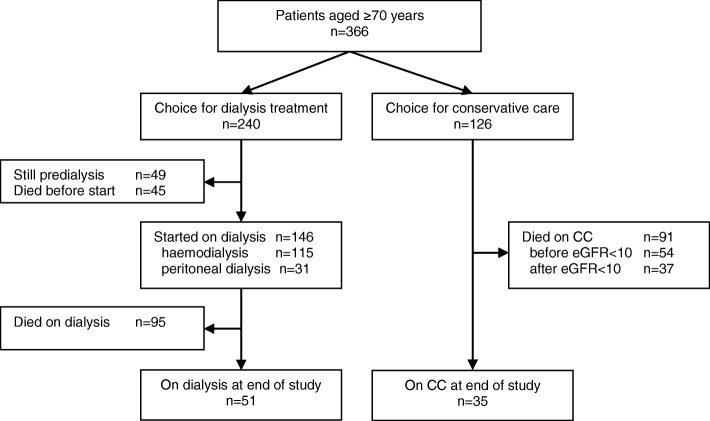
Overall flow of patients ≥70 years with advanced chronic kidney disease (stage 4/5). Fifteen patients changed their original choice in favour of dialysis to conservative care, and four changed from conservative care to dialysis. Analyses were based on the original treatment choice. CC, conservative care; eGFR, estimated glomerular filtration rate (mL/min/1.73m^2^)

Overall, we included 366 patients: 240 choosing dialysis, and 126 choosing CC (Fig. 1). Few patients changed their treatment choice: fifteen changed their original choice in favour of dialysis into CC, and four from CC into dialysis. Six patients underwent kidney transplantation, five after dialysis initiation (censored at date of transplantation). Baseline characteristics are shown in Table 1. Compared to the dialysis group, CC patients were older, more often female, and their eGFR at treatment decision was higher. There were no differences in Davies comorbidity score, primary renal diagnosis, body mass index, serum albumin, and C-reactive protein level.

**Table 1:** Baseline characteristics of patients choosing dialysis or conservative care, determined at treatment decision

	Dialysis (*n* = 240)	Conservative care (*n* = 126)	*P* value
Age (years), mean (SD)	76.2 (4.4)	82.6 (4.5)	< 0.001
Aged ≥80 years	55 (23%)	93 (74%)	< 0.001
Sex (female)	80 (33%)	58 (46%)	0.02
Davies comorbidity score			0.73
No comorbidity (score = 0)	27 (11%)	11 (9%)	
Intermediate comorbidity (score = 1 or 2)	142 (59%)	75 (59%)	
Severe comorbidity (score ≥ 3)	71 (30%)	40 (32%)	
Primary renal diagnoses			0.12
Renal vascular disease	82 (34%)	58 (46%)	
Diabetes mellitus	40 (17%)	16 (13%)	
Hypertension	21 (9%)	7 (6%)	
Pyelonephritis	5 (2%)	5 (4%)	
Polycystic kidneys	7 (3%)	1 (1%)	
Glomerulonephritis	7 (3%)	0 (0%)	
Cause unknown	58 (24%)	30 (24%)	
Other	20 (8%)	9 (7%)	
Body mass index (kg/m^2^), mean (SD)	27.0 (4.5; *n* = 196)	26.2 (4.8; *n* = 98)	0.16
Albumin (g/l), mean (SD)	39.2 (4.6; *n* = 186)	38.8 (3.5; *n* = 109)	0.42
C-reactive protein (nmol/l), median (IQR)	47.6 (28.6–95.2; *n* = 173)	47.6 (28.6–123.8; *n* = 93)	0.12
eGFR at treatment decision (mL/min/1.73m^2^), mean (SD)	13.3 (4.3)	15.6 (5.0)	< 0.001
Time of eGFR decline from < 20 to < 15 mL/min/1.73m^2^(days), median (IQR)	238 (52–461; *n* = 188)	212 (0–564; *n* = 80)	0.55
Time from treatment decision to dialysis initiation (days), median (IQR)	146 (48–437; *n* = 146)		
eGFR at start of dialysis (mL/min/1.73m^2^), mean (SD)	8.4 (2.9; *n* = 146)		

Values are numbers (percentages) unless stated otherwise

*eGFR* estimated glomerular filtration rate, *IQR* interquartile range, *SD* standard deviation

### Survival

**Fig. 2:**
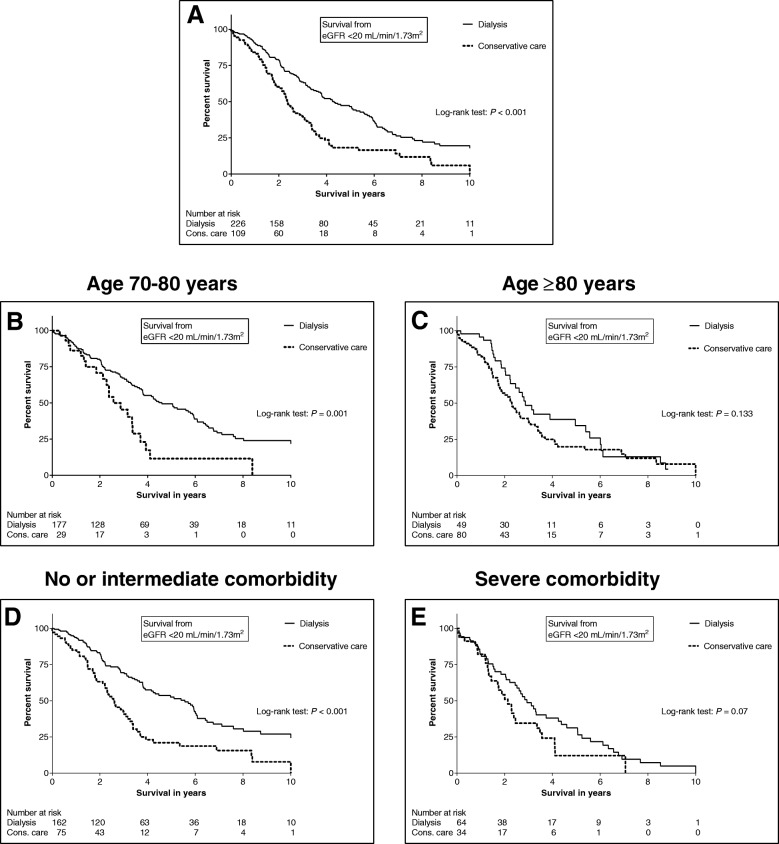
Kaplan-Meier survival curves comparing patients ≥70 years choosing dialysis or conservative care, from eGFR < 20 mL/min/1.73m^2^: overall comparison of both groups (part **a**; median survival: 4.3 [2.1–7.6] versus 2.4 [1.4–3.7] years); after stratification of age (**b** and **c**); after stratification of Davies comorbidity scores with no and intermediate comorbidity taken together versus severe comorbidity (**d** and **e**). The total number of included patients in this analysis was lower because some were referred after eGFR < 20 mL/min/1.73m^2^. See Additional files 2, 3 and 4 for the survival curves from the other starting points (treatment decision, eGFR < 15 and < 10 mL/min/1.73m^2^)

The dialysis group lived approximately twice as long compared to the CC group (Fig. 2a; median survival from eGFR < 20 mL/min/1.73m^2^: 4.3 [2.1–7.6] versus 2.4 [1.4–3.7] years, *P* <  0.001). However, this significant survival advantage of the dialysis group disappeared in patients aged ≥80 years (Fig. 2b, c; median survival: 2.9 [1.9–6.0] versus 2.3 [1.3–3.7] years, *P* = 0.13). The survival advantage of the dialysis group also lost significance in patients aged ≥70 years with Davies comorbidity scores ≥3 (severe comorbidity) (Fig. 2d, e; median survival: 2.9 [1.5–5.2] versus 2.1 [1.3–3.6] years, *P* = 0.07). Similar results were found using the other starting points (treatment decision, eGFR < 15 and < 10 mL/min/1.73m^2^): the significant survival benefit of the dialysis group diminished or disappeared in patients aged ≥80 years or with severe comorbidity (see Additional files 2, 3 and 4). Adjusted multivariable Cox regression analysis confirmed the observations that increasing age and severe comorbidity were independently associated with higher mortality (Table 2).

**Table 2:** Multivariable Cox proportional hazards model of survival in 240 patients choosing dialysis and 126 patients choosing conservative care, calculated from treatment decision

	Hazard ratio	95% CI for Hazard Ratio	*P* value
Age (years)	1.04	1.01 to 1.07	0.02
Female vs. Male	0.74	0.56 to 0.98	0.04
Davies comorbidity score			< 0.001
Intermediate comorbidity vs. No comorbidity	1.79	1.04 to 3.07	
Severe comorbidity vs. No comorbidity	3.48	1.99 to 6.11	
Conservative care vs. Dialysis	1.67	1.19 to 2.35	0.003

*CI* confidence interval, *vs* versus

### HRQOL

99 (77%) of 128 eligible patients consented for HRQOL assessment. Main reasons for non-participation were assumed response burden (*n* = 9), or unknown (17 non-responders). Three patients were excluded from analysis because of too many missings, leaving 96 patients (baseline characteristics: see Additional file 5). Of 34 dialysis patients, 26 were on HD, and 8 on PD.

Table 3 shows the results on PCS and MCS, and three kidney disease-specific domains of KDQOL-SF™ (all domain scores: see Additional file 6). No significant differences were found between dialysis patients and CC patients on PCS, MCS, symptoms, and effects of kidney disease. Dialysis patients scored worst on burden of kidney disease. Compared to patients not yet started on dialysis, CC patients scored significantly lower on PCS, MCS, symptoms, and effects of kidney disease on daily life, while no differences were observed on burden of kidney disease. After adjustment, multiple linear regression models confirmed the observations on PCS and MCS (Table 4).

**Table 3:** Physical and mental component summary scores^a^, and three kidney disease-specific domain scores^a^ from KDQOL-SF™

	Not yet started on dialysis (*n* = 39)	Started on dialysis (*n* = 34)	Conservative care (*n* = 23)	*P* value
Physical Component Summary score, mean (SD)	56.0 (20.6)	48.1 (20.9)	40.2 (16.2)	1: < 0.01^b^ 2: 0.14^c^ 3: 0.11^d^
Mental Component Summary score, mean (SD)	68.5 (17.2)	62.0 (22.0)	54.2 (19.7)	1: < 0.01 2: 0.18 3: 0.16
Kidney disease-specific symptoms and problems, median (IQR)	86.4 (68.2–88.6)	83.3 (70.6–89.6)	72.6 (61.4–83.0)	1: 0.03 2: 0.05 3: 0.81
Effects of kidney disease on daily life, median (IQR)	92.9 (78.6–96.4)	85.7 (67.9–96.4)	82.7 (58.9–90.2)	1: 0.03 2: 0.35 3: 0.26
Burden of kidney disease, median (IQR)	75.0 (56.3–93.8)	43.8 (25.0–62.5)	75.0 (56.3–81.3)	1: 0.70 2: 0.001 3: < 0.001

*IQR* interquartile range, *KDQOL-SF™*, Kidney Disease Quality of Life Short Form, *SD* standard deviation

^a^= Scores range between 0 and 100; higher scores indicate better health-related quality of life

^b^= Not yet started on dialysis versus Conservative care;

^c^= Started on dialysis versus Conservative care;

^d^= Not yet started on dialysis versus Started on dialysis

**Table 4:** Multiple linear regression models of the PCS and MCS from KDQOL-SF™ in patients choosing dialysis but not yet started on dialysis (*n* = 39), in patients started on dialysis (*n* = 34), and in patients choosing conservative care (*n* = 23)

	B	95% CI for B	*P* value
Physical Component Summary score^a^			
Constant	41.31	32.54 to 50.09	
Female vs. Male	−10.01	−18.28 to − 1.73	0.02
Interviewer-administration vs. Self-administration	14.23	5.63 to 22.84	0.001
Not yet started on dialysis vs. Conservative care	15.24	5.46 to 25.03	0.003
Started on dialysis vs. Conservative care	1.58	−8.87 to 12.04	0.76
Mental Component Summary score^b^			
Constant	48.82	41.30 to 56.34	
Interviewer-administration vs. Self-administration	20.49	12.41 to 28.57	< 0.001
Not yet started on dialysis vs. Conservative care	16.03	6.90 to 25.16	0.001
Started on dialysis vs. Conservative care	2.01	−7.67 to 11.69	0.68

*CI* confidence interval, *KDQOL-SF™* Kidney Disease Quality of Life Short Form, *MCS* Mental Component Summary, *PCS* Physical Component Summary, *vs* versus

^a^= Physical Component Summary score model: R^2^ = 0.22, F(4,91) = 6.36, *P* <  0.001. Results were similar when additionally adjusted for age and Davies comorbidity score

^b^= Mental Component Summary score model: R^2^ = 0.28, F(3,91) = 11.77, *P* <  0.001. Results were similar when additionally adjusted for age, sex, and Davies comorbidity score

### Treatment burden

358 patients were included in the analyses on treatment burden: 233 in the dialysis group (602.1 person years), and 125 in the CC group (198.7 person years), excluding 8 who were lost to follow-up due to referall to other centers. Of 233 patients in the dialysis group, 140 started dialysis.

Table 5 shows the results for treatment burden. CC patients had significant lower treatment burden compared with the dialysis group: less outpatient visits, admissions, and in-hospital days, resulting in more hospital free days (part A). The adjusted incidence rate ratios confirmed these findings. In the dialysis group, the overall incidence rate of in-center haemodialysis days calculated from treatment decision was 60.6 days per person year (hence, also capturing time between treatment decision and dialysis initiation). The number of in-center haemodialysis days calculated from start of dialysis in haemodialysis patients only (*n* = 110) was 150.3 days per person year, resulting in less hospital free days (part B).

**Table 5:** Outcomes on treatment burden. Annual treatment burden of patients choosing dialysis versus patients choosing conservative care, measured from treatment decision (part A). Annual treatment burden of patients started on dialysis—a subgroup of all patients choosing dialysis—measured from start of dialysis (part B)

A – *from treatment decision*	Dialysis (*n* = 233) Incidence rate	Conservative care (*n* = 125) Incidence rate	Incidence rate ratio^b^ (95% CI)	*P* value
Outpatient visits per person year	11.1	6.6	0.63 (0.53 to 0.75)	< 0.001
Admissions per person year	2.0	1.1	0.57 (0.42 to 0.78)	< 0.001
In-hospital days per person year	10.8	6.0	0.43 (0.28 to 0.66)	< 0.001
In-center haemodialysis days per person year	60.6	–	–	–
Hospital free days per person year^a^	282.7	352.7	1.15 (1.09 to 1.21)	< 0.001
B – *from start of dialysis*	Haemodialysis (*n* = 110) Incidence rate	Peritoneal dialysis (*n* = 30) Incidence rate	Incidence rate ratio^c^ (95% CI)	*P* value
Outpatient visits per person year	9.0	15.2	1.80 (1.43 to 2.26)	< 0.001
Admissions per person year	2.4	2.3	1.03 (0.71 to 1.49)	0.88
In-hospital days per person year	14.6	14.8	1.46 (0.80 to 2.68)	0.22
In-center haemodialysis days per person year	150.3	–	–	–
Hospital free days per person year^a^	191.4	335.4	1.72 (1.65 to 1.80)	< 0.001

^a^= Hospital free days are calculated using the formula: 365.25 – (annual incidence rates of outpatient visits + in-hospital days + in-center haemodialysis days)

^b^= Adjusted for age, sex, Davies comorbidity score, and estimated glomerular filtration rate

^c^= Adjusted for age, sex, and Davies comorbidity score

### Treatment costs

Cost data were available for 262 patients: 162 choosing dialysis (380.7 person years), 100 choosing CC (153.2 person years). Of 162 patients choosing dialysis, 84 started dialysis. Baseline characteristics were similar compared with the overall cohort.

Table 6 shows the results on treatment costs incurred in our hospital, indicating significant lower costs in the CC group. The cost ratio—after adjustment for age, sex, Davies comorbidity score, and eGFR—was 0.43 (95% CI 0.28 to 0.67, *P* <  0.001). Results were similar in sensitivity analyses fitting generalised linear models with log-y or Poisson distribution.

**Table 6:** Treatment costs. Mean annual treatment costs of patients choosing dialysis versus patients choosing conservative care, measured from treatment decision (part A). Mean annual treatment costs of patients started on dialysis—a subgroup of all patients choosing dialysis—measured from start of dialysis (part B)

A *– from treatment decision*	Dialysis (*n* = 162)	Conservative care (*n* = 100)	Cost ratio^a^ (95% CI)	*P* value
Costs per person year, €	28,353	5861	0.43 (0.28 to 0.67)	< 0.001
B *– from start of dialysis*	Started on dialysis (*n* = 84; 64 on HD; 20 on PD)			
Costs per person year, €	54,907			

*HD* haemodialysis, *PD* peritoneal dialysis

^a^adjusted for age, sex, Davies comorbidity score, and estimated glomerular filtration rate

## Discussion

In this retrospective observational cohort study, we evaluated a combination of patient-relevant outcomes and treatment costs in one of the largest groups reported so far of older patients (≥70 years) with advanced CKD who have chosen to be treated with dialysis or CC. Patients choosing dialysis lived longer compared with patients choosing CC, but there was little or no significant survival advantage in patients aged ≥80 years or with severe comorbidity. In a smaller subset, no significant differences were observed in physical and mental health scores between patients on dialysis or CC, while dialysis patients scored worst on burden of kidney disease. Treatment burden was substantially lower in patients on CC, including less frequent outpatient visits, admissions, in-hospital days, and no in-center haemodialysis days, resulting in more hospital free days. CC patients incurred significantly lower treatment costs in our hospital. By carefully delivering patient-centered care with shared decision-making on treatment plan, value was generated for patients choosing CC and society by achieving similar outcomes at lower treatment burden and treatment costs.

To inform shared decision-making on dialysis or conservative care, data on patient-relevant outcomes are needed. Studies comparing older patients on dialysis and CC have been cohort or case-control studies, with the majority being retrospective, small, single-center, and from the United Kingdom [6–8]. Appropriate comparison of results across these studies is hindered by methodological issues including heterogeneous populations, allocation bias, and variation in definition of time points used in analysis. Whilst a randomized controlled trial offers a more ideal study design to compare outcomes between groups, such study design in this setting would pose difficult, if not impossible, ethical and practical dilemmas and has not been reported so far.

Survival has been studied most frequently, and is regarded as important outcome by kidney patients [16–19]. In general, all survival studies showed a significant survival advantage in the dialysis group, but there was little or no significant survival benefit when comparison was restricted to patients with high age or high comorbidity scores [6–8, 29, 30]. Best available evidence comes from a prospective study [31], the relatively largest retrospective study performed so far [32], and two smaller retrospective studies [33, 34]. We found similar results in a large Dutch population aged ≥70 years, comparable with our previous survival analysis (see Additional file 7) [9]. Due to differences between studies in design and findings, it is difficult to identify a consistent cut-off level for age or comorbidity score from which the survival advantage of dialysis is no longer significant. While a cut-off level could be useful to help identify which patients are likely to benefit from dialysis, there is great risk of oversimplifying decision-making, for example by overlooking relevant individual factors. It should also be noted that the results of the subgroup analyses based on age and comorbidity have to be interpreted with caution, as the patient numbers in the subgroups are lower. We think that the most appropriate conclusions on survival so far are that 1) increasing age and comorbidity are associated with decreasing survival benefit in patients choosing dialysis compared to choosing CC, and that 2) the survival advantage of dialysis is no longer significant in patients with the highest ages and severe comorbidity.

HRQOL is one of the most important outcomes to kidney patients [16–19, 35], but studies comparing HRQOL between dialysis and CC are limited [36]. HRQOL has mostly been assessed with the generic Short Form-36 (SF-36) [31, 37–40]. In general, CC patients are found to have similar PCS and MCS scores compared to patients on a dialysis pathway. Our results, although determined in small groups, are consistent with these findings. Two studies observed a lower PCS score at baseline in the CC group compared to patients on a dialysis pathway, but this difference can be explained by the significant group differences in age and comorbidity [31, 37]. In our study, there were no differences in comorbidity scores, and age differences were substantially smaller. The observed difference in PCS scores in one study [37] can also be explained by including predialysis and dialysis patients in one overall dialysis group. We found significant differences between these groups, and therefore believe that separating these dialysis subgroups is essential to perform meaningful comparisons on HRQOL. Kidney disease-specific domains of HRQOL have been assessed by only one study so far, using KDQOL-SF™ [40]. Seow et al. observed that dialysis initiation was associated with worse scores on burden of kidney disease, and effects of kidney disease on daily life [40]. We also observed the worst scores on burden of kidney disease in dialysis patients. To confirm the findings on HRQOL and truly inform decision-making, longitudinal HRQOL assessments—capturing generic *and* kidney disease-specific domains— in larger cohorts are needed.

Treatment burden is shown to be very relevant to kidney patients, particularly when considering dialysis or CC [16–19, 35, 41]. Only few studies have compared treatment burden so far, although detailed definitions are often missing—for example whether in-center haemodialysis days or outpatient visits were assessed [32, 42–45]. In general, patients on a dialysis pathway are observed to have higher hospitalization rates and to spend more time in hospital compared with CC patients. Our results are consistent with these findings while providing more insight in different domains of treatment burden. By doing so, we were able to estimate the number of hospital free days as summary measure. Although the burden of each domain could be experienced differently, hospital free time is shown to be one of most relevant outcomes to kidney patients and could be a major reason for older patients to choose CC instead of dialysis [17–19, 41, 46].

We assessed treatment costs to determine value of care. Whilst cost data will not help patients in their decision-making, insight is needed for other stakeholders like health policy makers and society. Unsurprisingly, we observed lower treatment costs in patients on CC. Only three previous studies compared costs in patients on dialysis or CC. All reported lower costs in the CC group [42, 47, 48], despite methodological issues [49]. Comprehensive economic evaluations are needed to confirm these results [49].

Our observational study addresses the knowledge gap on patient-relevant outcomes and value of care in older kidney patients treated with dialysis or CC, and will help inform decision-making on preferred treatment. Potential flaws are allocation bias and confounding by indication, both inherent to the non-random treatment decision. In our cohort, the CC group was substantially older compared with the dialysis group, whilst comorbidity—which often differs between both groups in other studies [6, 8]—was similar. We confirmed our findings in multivariable regression analyses with adjustment for several confounders. However, residual confounders might be missing. Bias could also be present due to the sample size, particularly in our HRQOL assessment. Our findings primarily stress the need for larger comparative studies focusing on more than survival only.

A methodological difficulty in outcome comparisons between dialysis and conservative care is to define equivalent time points for both treatment pathways. Theoretically, the best possible starting point would be the start of dialysis, representing the moment that each group starts receiving their specific treatment. However, this time point is not applicable in CC patients and identifying an equivalent moment of a putative or assumed dialysis start is difficult [8]. The use of other time points means that observed outcomes are not the exclusive result of received treatment but rather are associated with being in the group who chose dialysis or CC. Like many other studies [31, 37, 50–52], we used time of treatment decision as starting point. A concern about this time point is that lead time bias might be present, indicated in our study by a significant difference in eGFR at treatment decision between both patient groups. Therefore, we also used three time points reaching a threshold eGFR in survival analyses, and adjusted for eGFR at treatment decision in the multivariable regression analyses of survival, treatment burden, and treatment costs.

As data on treatment burden and treatment costs were only available from our hospital, potential data from other hospitals, primary care, social services, nursing homes, and on out-of-hospital medication are missing. Such data are required to fully assess whether value has been increased by taking into account the entire care pathway. We see our evaluation as a first step.

## Conclusions

We studied a combination of patient-relevant outcomes and treatment costs, and showed that patients ≥70 years choosing CC instead of dialysis—particularly the oldest old and those with severe comorbidity—achieved similar survival and HRQOL outcomes at lower treatment burden and treatment costs. We believe CC to be a viable treatment option in older CKD patients. With CC, value of care can be generated: for patients in terms of patient-relevant outcomes in balance with treatment burden; for society in terms of patient-relevant outcomes per monetary unit spent. These findings emphasize the need to openly discuss all treatment options including CC to align treatment plan with what matters to older patients with advanced CKD.

## Additional files

Additional file 1: Methods. Details on the Davies comorbidity score, KDQOL-SF™, and treatment burden outcomes. (PDF 16 kb)

Additional file 2: **Figure S1.** Kaplan-Meier survival curves comparing patients ≥70 years choosing dialysis or conservative care, from treatment decision. (PDF 87 kb)

Additional file 3: **Figure S2.** Kaplan-Meier survival curves comparing patients ≥70 years choosing dialysis or conservative care, from eGFR < 15 mL/min/1.73m^2^. (PDF 91 kb)

Additional file 4: **Figure S3.** Kaplan-Meier survival curves comparing patients ≥70 years choosing dialysis or conservative care, from eGFR < 10 mL/min/1.73m^2^. (PDF 76 kb)

Additional file 5: **Table S1.** Baseline characteristics of patients included in the assessment of HRQOL. (PDF 12 kb)

Additional file 6: **Table S2.** All KDQOL-SF™ domain scores in patients choosing dialysis but not yet started on dialysis, in patients started on dialysis, and in patients on conservative care. (PDF 7 kb)

Additional file 7: Discussion. Comparison between the current and previous results on survival. (PDF 11 kb)

## Abbreviations

CC: Conservative care
CI: Confidence interval
CKD: Chronic kidney disease
eGFR: Estimated glomerular filtration rate
HD: Haemodialysis
HRQOL: Health-related quality of life
KDQOL-SF™: Kidney disease quality of life short form
MCS: Mental component summary
PCS: Physical component summary
PD: peritoneal dialysis
SF-36: Short form-36
SPSS: Statistical package for the social science
