# Supplementary material for: Value-based evaluation of dialysis versus conservative care in older patients with advanced chronic kidney disease: a cohort study
Source: BMC Nephrol. 2018 Aug 16;19:205. doi: 10.1186/s12882-018-1004-4 (PMC6097302; doi:10.1186/s12882-018-1004-4)
Supplement: Supplementary file 1 — Methods. Details on the Davies comorbidity score, KDQOL-SF™, and treatment burden outcomes. (PDF 16 kb) [file 12882_2018_1004_MOESM1_ESM.pdf]

ADDITIONAL FILE 1: Additional Methods

Davies comorbidity score

The Davies comorbidity score is based on the presence of seven comorbid conditions [1], including: ischemic heart disease, left ventricular dysfunction, peripheral vascular disease, malignancy, diabetes mellitus, systemic collagen vascular disease, and other significant disorders like chronic obstructive pulmonary disease. Each condition is scored with one point, which is used to categorize three groups: no comorbidity (Davies comorbidity score = 0), intermediate comorbidity (Davies comorbidity score = 1 or 2), and severe comorbidity (Davies comorbidity score ≥ 3).

KDQOL-SF™

The Kidney Disease Quality of Life Short Form (KDQOL-SF™) covers both generic and kidney disease-specific domains [2, 3]. The eight generic domains—which are the same domains as in the commonly assessed Short-Form 36 Health Survey (SF-36) developed by the RAND Corporation [4]—are used to generate a Physical Component Summary (PCS) score and a Mental Component Summary (MCS) score. PCS and MCS are shown to be most appropriate in renal patients on conservative care rather than the scores on the individual generic domains [5]. The kidney disease-specific domains included: symptoms/problems of kidney disease, effects of kidney disease on daily life, burden of kidney disease, cognitive function, quality of social interaction, sleep, and social support. Although the disease-specific domains have only been validated in patients on dialysis, RAND suggests to use it in renal patients not on dialysis as well [6]. In our study, four disease-specific domains were not measured, including: work status, sexual function, dialysis staff encouragement, and patient satisfaction with care. Work status was not included since most patients were not working. Sexual function was excluded because of expected missing variables, which is described in older patients [3]. Dialysis staff encouragement, and patient satisfaction with care were excluded because they are not applicable in patients not on dialysis. The KDQOL-SF™ items were coded and scored according to the manual [7]. If more than 50% of items needed to calculate a domain score were missing, the corresponding domain score was regarded as missing. Interviewer-administration is allowed. Scores range between 0 and 100; higher scores indicate better health-related quality of life (HRQOL).

Treatment burden outcomes

In determining the number of outpatient visits, two or more outpatient visits on the same day were counted as one outpatient visit. In the numbers of admissions and in-hospital days, both planned and unplanned admissions were included without additional criteria on minimum length of stay. Hence, hospital stays shorter than 24 hours (in general planned short-stay admissions) were included as well and were counted as one day. In patients on haemodialysis, the number of in-center haemodialysis days was estimated by multiplying the total number of weeks on haemodialysis (from start of haemodialysis until death or end of study) with three, knowing that the vast majority of patients were

dialysed three times per week. In-center haemodialysis days were not counted on in-hospital days to prevent duplications.

References:

1. Davies SJ, Russell L, Bryan J, Phillips L, Russell GI. Comorbidity, urea kinetics, and appetite in continuous ambulatory peritoneal dialysis patients: their interrelationship and prediction of survival. Am J Kidney Dis. 1995;26(2):353-61.
2. Hays RD, Kallich JD, Mapes DL, Coons SJ, Carter WB. Development of the kidney disease quality of life (KDQOL) instrument. Qual Life Res. 1994;3(5):329-38.
3. Korevaar JC, Merkus MP, Jansen MA, Dekker FW, Boeschoten EW, Krediet RT, et al. Validation of the KDQOL-SF: a dialysis-targeted health measure. Qual Life Res. 2002;11(5):437-47.
4. Ware JE, Sherbourne CD. The MOS 36-item short-form health survey (SF-36). I. Conceptual framework and item selection. *Med Care* 1992;30(6):473-83.
5. Erez G, Selman L, Murtagh FE. Measuring health-related quality of life in patients with conservatively managed stage 5 chronic kidney disease: limitations of the Medical Outcomes Study Short Form 36: SF-36. *Qual Life Res* 2016;25(11):2799-809.
6. RAND Health. KDQOL Frequently Asked Questions. Available from: http://www.rand.org/health/surveys_tools/kdqol/faq.html; Accessed: June, 2017.
7. Hays RD, Kallich JD, Mapes DL, et al. Kidney Disease Quality of Life Short Form (KDQOL-SF^{TM}), Version 1.3: A Manual for Use and Scoring. Santa Monica, CA: RAND,1995.
