# Supplementary material for: Value-based evaluation of dialysis versus conservative care in older patients with advanced chronic kidney disease: a cohort study
Source: BMC Nephrol. 2018 Aug 16;19:205. doi: 10.1186/s12882-018-1004-4 (PMC6097302; doi:10.1186/s12882-018-1004-4)
Supplement: Supplementary file 2 — Figure S1. Kaplan-Meier survival curves comparing patients ≥70 years choosing dialysis or conservative care, from treatment decision. (PDF 87 kb) [file 12882_2018_1004_MOESM2_ESM.pdf]

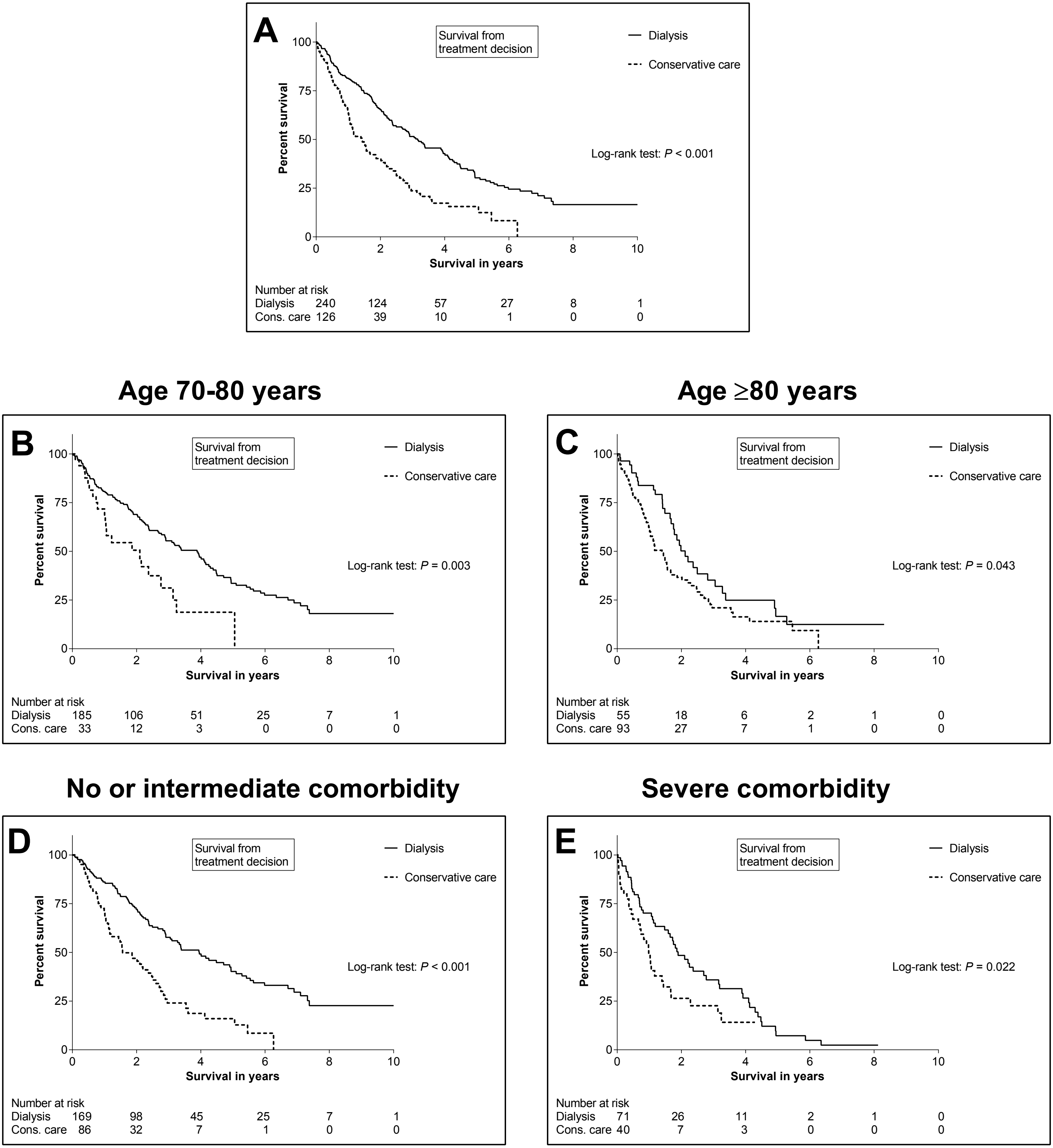

Additional Figure 1. Kaplan-Meier survival curves comparing patients ≥70 years choosing dialysis or conservative care, from treatment decision: overall comparison of both groups (part A; median survival:3.2 [1.5-6.0] versus 1.4 [0.7-2.9] years); after stratification of age (B and C); after stratification of Davies comorbidity scores with no and intermediate comorbidity taken together versus severe comorbidity (D and E).
