# Supplementary material for: Value-based evaluation of dialysis versus conservative care in older patients with advanced chronic kidney disease: a cohort study
Source: BMC Nephrol. 2018 Aug 16;19:205. doi: 10.1186/s12882-018-1004-4 (PMC6097302; doi:10.1186/s12882-018-1004-4)
Supplement: Supplementary file 3 — Figure S2. Kaplan-Meier survival curves comparing patients ≥70 years choosing dialysis or conservative care, from eGFR < 15 mL/min/1.73m2. (PDF 91 kb) [file 12882_2018_1004_MOESM3_ESM.pdf]

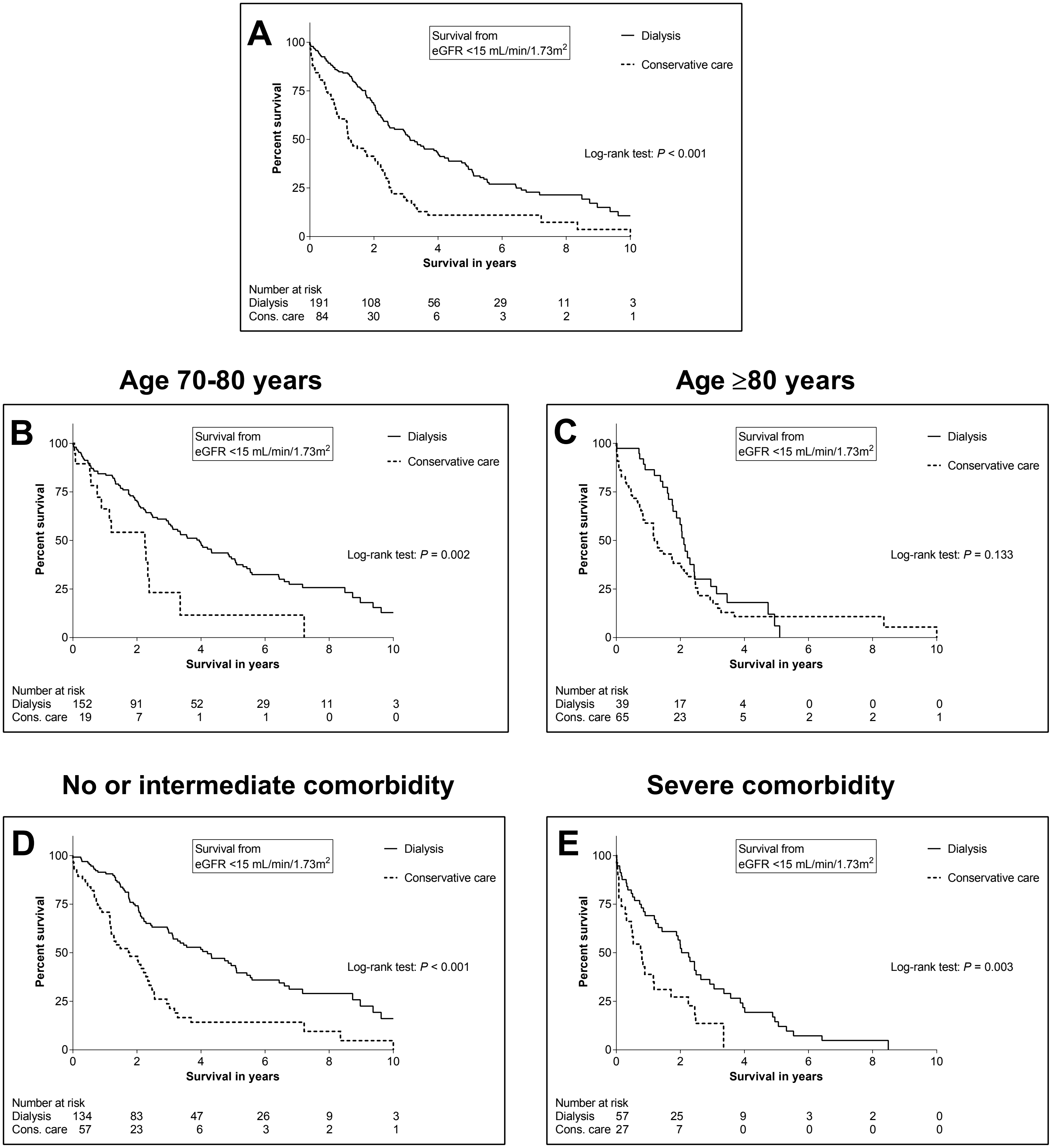

Additional Figure 2. Kaplan-Meier survival curves comparing patients ≥70 years choosing dialysis or conservative care, from eGFR <15 mL/min/1.73m^2: overall comparison of both groups (part A; median survival:3.1 [1.7-6.4] versus 1.3 [0.5-2.5] years); after stratification of age (B and C); after stratification of Davies comorbidity scores with no and intermediate comorbidity taken together versus severe comorbidity (D and E). The total number of patients included in this analysis was lower because some patients died before eGFR dropped <15 mL/min/1.73m^2.
