# Supplementary material for: Value-based evaluation of dialysis versus conservative care in older patients with advanced chronic kidney disease: a cohort study
Source: BMC Nephrol. 2018 Aug 16;19:205. doi: 10.1186/s12882-018-1004-4 (PMC6097302; doi:10.1186/s12882-018-1004-4)
Supplement: Supplementary file 5 — Table S1. Baseline characteristics of patients included in the assessment of HRQOL. (PDF 12 kb) [file 12882_2018_1004_MOESM5_ESM.pdf]

ADDITIONAL FILE 5:

Additional Table 1. Baseline characteristics of patients included in the assessment of HRQOL.

	**Not yet started on dialysis** (n=39)	**Started on dialysis** (n=34)	**Conservative care** (n=23)	***P* value**
Mean (SD) age (years)	79.8 (5.1)	80.1 (3.3)	83.8 (5.0)	1: 0.004[a]<br>2: 0.001[b]<br>3: 0.78[c]
Aged ≥80 years	14 (36%)	7 (21%)	15 (65%)	1: 0.03<br>2: 0.11<br>3: 0.56
Sex (female)	12 (31%)	8 (24%)	11 (48%)	1: 0.18<br>2: 0.06<br>3: 0.49
Davies comorbidity score				1: 0.85<br>2: 0.87<br>3: 0.83
No comorbidity (score = 0)	5 (13%)	3 (9%)	2 (9%)	
Intermediate comorbidity (score = 1 or 2)	23 (59%)	20 (59%)	15 (65%)	
Severe comorbidity (score ≥ 3)	11 (28%)	11 (32%)	6 (26%)	
Primary renal diagnosis				1: 0.30<br>2: 0.05<br>3: 0.20
Renal vascular disease	15 (39%)	10 (29%)	8 (35%)	
Diabetes mellitus	2 (5%)	5 (15%)	0 (0%)	
Hypertension	2 (5%)	5 (15%)	0 (0%)	
Pyelonephritis	0 (0%)	1 (3%)	3 (13%)	
Polycystic kidneys	1 (3%)	3 (9%)	1 (4%)	
Glomerulonephritis	1 (3%)	2 (6%)	0 (0%)	
Cause unknown	11 (28%)	6 (18%)	6 (26%)	
Other	7 (18%)	2 (6%)	5 (22%)	
Mean (SD) eGFR (mL/min/1.73 m²) at HRQOL assessment	16.2 (5.4)		16.4 (5.6)	1: 0.86
Median (IQR) time in years from treatment choice to HRQOL assessment	1.1 (0.6 – 1.7)	2.9 (1.0 – 5.5)	1.3 (0.3 – 2.6)	1: 0.62<br>2: 0.001<br>3: <0.001
Median (IQR) time in years from dialysis start to HRQOL assessment		1.8 (0.5 – 4.4)		
Interviewer-administration of KDQOL-SF™	7 (18%)	18 (53%)	6 (26%)	1: 0.45<br>2: 0.04<br>3: 0.002

Values are numbers (percentages) unless stated otherwise.

eGFR, estimated glomerular filtration rate; HRQOL, health-related quality of life; IQR, interquartile range; SD, standard deviation.

[a]= Not yet started on dialysis *versus* Conservative care;

[b]= Started on dialysis *versus* Conservative care;

[c]= Not yet started on dialysis *versus* Started on dialysis.
