# Supplementary material for: Value-based evaluation of dialysis versus conservative care in older patients with advanced chronic kidney disease: a cohort study
Source: BMC Nephrol. 2018 Aug 16;19:205. doi: 10.1186/s12882-018-1004-4 (PMC6097302; doi:10.1186/s12882-018-1004-4)
Supplement: Supplementary file 6 — Table S2. All KDQOL-SF™ domain scores in patients choosing dialysis but not yet started on dialysis, in patients started on dialysis, and in patients on conservative care. (PDF 7 kb) [file 12882_2018_1004_MOESM6_ESM.pdf]

ADDITIONAL FILE 6:

Additional Table 2. All KDQOL-SF^{TM} domain scores in patients choosing dialysis but not yet started on dialysis, in patients started on dialysis, and in patients on conservative care.

	**Not yet started on dialysis** (n=39)	**Started on dialysis** (n=34)	**Conservative care** (n=23)	***P* value**
Generic (SF-36) domains				
Physical function	50.0 (30.0 – 75.0)	30.0 (10.0 – 70.0)	25.0 (5.0 – 45.0)	1: <0.001[a]<br>2: 0.25[b]<br>3: 0.02[d]
Role function - physical	50.0 (0.0 - 100.0)	25.0 (0.0 - 75.0)	25.0 (0.0 – 75.0)	1: 0.63<br>2: 0.83<br>3: 0.41
Bodily pain	80.0 (45.0 – 100.0)	73.8 (45.0 – 100.0)	57.5 (32.5 – 90.0)	1: 0.10<br>2: 0.06<br>3: 0.90
General health	50.0 (38.8 – 65.0)	37.5 (25.0 – 60.0)	35.0 (25.0 – 45.0)	1: <0.005<br>2: 0.57<br>3: 0.07
Vitality	60.0 (45.0 – 80.0)	57.5 (35.0 – 75.0)	45.0 (25.0 – 55.0)	1: <0.005<br>2: 0.03<br>3: 0.34
Social function	87.5 (75.0 – 100.0)	62.5 (50.0 – 100.0)	62.5 (37.5 – 87.5)	1: 0.01<br>2: 0.69<br>3: 0.01
Role function - emotional	100.0 (0.0 – 100.0)	100.0 (0.0 – 100.0)	100.0 (0.0 - 100.0)	1: 0.81<br>2: 0.79<br>3: 0.95
Mental health	84.0 (80.0 – 92.0)	84.0 (67.0 – 92.0)	76.0 (60.0 – 80.0)	1: 0.001<br>2: 0.03<br>3: 0.59
Kidney disease-specific domains				
Symptoms / problems	86.4 (68.2 – 88.6)	83.3 (70.6 – 89.6)	72.6 (61.4 – 83.0)	1: 0.03<br>2: 0.05<br>3: 0.81
Effects of kidney disease on daily life	92.9 (78.6 – 96.4)	85.7 (67.9 – 96.4)	82.7 (58.9 – 90.2)	1: 0.03<br>2: 0.35<br>3: 0.26
Burden of kidney disease	75.0 (56.3 – 93.8)	43.8 (25.0 – 62.5)	75.0 (56.3 – 81.3)	1: 0.70<br>2: 0.001<br>3: <0.001
Cognitive function	86.7 (73.3 – 100.0)	86.7 (71.7 – 93.3)	73.3 (60.0 – 86.7)	1: 0.01<br>2: 0.09<br>3: 0.33
Quality of social interaction	93.3 (80.0 – 100.0)	93.3 (78.3 – 100.0)	80.0 (66.7 – 93.3)	1: 0.03<br>2: 0.04<br>3: 0.78
Sleep	70.0 (61.9 – 80.6)	66.3 (58.8 – 80.6)	65.0 (47.5 – 75.0)	1: 0.19<br>2: 0.66<br>3: 0.41
Social support	100.0 (83.3 – 100.0)	100.0 (79.2 – 100.0)	83.3 (66.7 – 100.0)	1: 0.53<br>2: 0.29<br>3: 0.52

Values are presented as median (interquartile range). Higher scores (0-100) indicate better quality of life.

[a]= Not yet started on dialysis *versus* Conservative care; [b]= Started on dialysis *versus* Conservative care;

[c]= Not yet started on dialysis *versus* Started on dialysis.
