# Supplementary material for: Value-based evaluation of dialysis versus conservative care in older patients with advanced chronic kidney disease: a cohort study
Source: BMC Nephrol. 2018 Aug 16;19:205. doi: 10.1186/s12882-018-1004-4 (PMC6097302; doi:10.1186/s12882-018-1004-4)
Supplement: Supplementary file 7 — Discussion. Comparison between the current and previous results on survival. (PDF 11 kb) [file 12882_2018_1004_MOESM7_ESM.pdf]

ADDITIONAL FILE 7: Additional Discussion

Comparison between current and previous results on survival

Our current survival analysis was an extension of a previous assessment [1], having data of an enlarged cohort with longer follow-up time. Most results were similar, though we observed few differences compared to the previous analysis in the results of subgroup analysis based on age or Davies comorbidity score, depending on which starting point was used. The following differences were observed: the dialysis group remained their statistical survival advantage, though diminished, in patients ≥80 years when using time of treatment decision, or eGFR <10 mL/min/1.73m^2 as starting point; and in patients with severe Davies comorbidity scores, we now observed no longer a statistical survival benefit in the dialysis group using eGFR <20 mL/min/1.73m^2 as starting point. These differences between our current and previous survival analysis indicate a level of uncertainty. The smaller sample sizes in the subgroup analyses are a likely explanation. Further confirmation in larger cohorts is needed. Importantly, the results of the adjusted survival analysis using Cox proportional hazard models (see Table 2) are consistent with our previous results, showing that increasing age and higher Davies comorbidity scores are important predictors of mortality regardless of treatment pathway.

References:

1. Verberne WR, Geers AB, Jellema WT, Vincent HH, van Delden JJ, Bos WJ. Comparative Survival among Older Adults with Advanced Kidney Disease Managed Conservatively Versus with Dialysis. Clin J Am Soc Nephrol. 2016;11(4):633-40.
